# Data on vildagliptin and vildagliptin plus metformin combination in type-2 diabetes mellitus management

**DOI:** 10.6026/97320630017413

**Published:** 2021-03-31

**Authors:** Sambit Das, AK Gupta, Biplab Bandyopadhyaya, B Harish Darla, Vivek Arya, Mahesh Abhyankar, Santosh Revankar

**Affiliations:** 1 Endeavour Clinics, Bhubaneswar, India; 2 Rahas Medical Store Hospital, Lucknow, India; 3 Thyroid, Diabetes and Hormone Care, Indore, India; 4 Darla's Healthcare, Mysore, India; 5 Center for Endocrine and Diabetes, Ahmedabad, India; 6 USV Pvt. Ltd., Mumbai, India

**Keywords:** Antidiabetic therapy, DPP4i, glycemic control, hypertension

## Abstract

It is of interest to evaluate the clinical effectiveness and safety of vildagliptin as monotherapy and combination therapy of vildagliptin and metformin for the management of type 2 diabetes mellitus (T2DM) patients in Indian settings. The study included patients with T2DM (aged >18 years) receiving vildagliptin monotherapy and vildagliptin in combination with metformin therapy of various strengths. Data related to demographics, risk factors, medical history, glycated hemoglobin (HbA1c) levels, and medical therapies were retrieved from medical records. Out of 9678 patients (median age, 52.0 years), 59.1% were men. A combination of vildagliptin and metformin (50/500 mg) was the most commonly used therapy (54.8%), and the median duration of therapy was 24.0 months. The predominant reason for selecting vildagliptin therapy was to improve HbA1c levels (87.8%). A total of 87.5% of patients required dosage up-titration. Vildagliptin therapy was used in patients with T2DM and associated complications (peripheral neuropathy, CAD, nephropathy, retinopathy, autonomous neuropathy, stroke/TIA, and peripheral artery disease). Among 5175 patients who experienced body weight changes, a majority of patients showed a loss of weight (68.6%). The target glycemic control was achieved in 95.3% of patients. The mean HbA1c levels were significantly decreased post-treatment (mean change: 1.34%; p<0.001). Adverse events were reported in 0.4% of patients. Physicians rated the majority of patients as good to excellent on the global evaluation of efficacy and tolerability scale (98.9%, each). Vildagliptin as monotherapy and combination therapy of vildagliptin and metformin was an effective therapy in reducing HbA1c helps in achieving target glycemic control, and was well tolerated in Indian patients with T2DM continuum.

## Background

The poor glycemic control, long duration of illness, and the ethnicity of the population contribute to the increased susceptibility to diabetes associated complications [1]. Indian individuals with T2DM are highly susceptible to the risk of developing macrovascular complications with a 40% higher risk of mortality due to cardiovascular diseases, as compared to the White populations [2-4]. Moreover, the prevalence of comorbidities in Indian patients with diabetes is high along with peripheral vascular disease, hypertension, ocular diseases, and dyslipidemia as the most common comorbidities [5-8]. Therefore, early diagnosis of diabetes and improved glycemic control will aid in alleviating the risk factors of these patients. The American Diabetes Association (ADA), the European Association for the Study of Diabetes (EASD), and the India-specific diabetes management guidelines recommend the use of metformin along with lifestyle changes as first-line therapy for diabetes management [9-11]. Targeted glycemic levels may not be achieved by metformin or other oral antidiabetic drugs (OADs) monotherapy. Hence, considering diabetes as a progressive disease, combination therapies of metformin with other oral antidiabetic drugs (OADs) are recommended [9- 11]. Moreover, if patients fail to achieve target glycemic levels with monotherapy, the combination therapies of metformin with other OADs and/or insulin are recommended [9-11]. Vildagliptin is a second-generation dipeptidyl peptidase-4 (DPP-4) inhibitor [12] and evidence suggests potential mechanisms of synergy between metformin and vildagliptin [13,14]. The clinical efficacy and safety of vildagliptin monotherapy or in combination with metformin have been demonstrated in several studies. Globally, the effectiveness, tolerability, and low discontinuation rates of vildagliptin monotherapy and combination therapy of vildagliptin and metformin are reported in several real-world studies [15- 17]. However, there is a need for evidence from India demonstrating the overall clinical benefits of glycemic control and weight reduction with vildagliptin monotherapy [18] or vildagliptin and metformin combination therapy for diabetes [19,20]. Therefore, it is of interest to document the treatment patterns, clinical effectiveness, and safety profile of vildagliptin monotherapy, vildagliptin, and metformin combination therapy for the management of the T2DM continuum in India.

## Methods

This retrospective, multi-centric, observational real-world study was conducted across 365 Indian healthcare centers having medical records of adult patients with T2DM who had received vildagliptin alone or as an add-on to metformin therapy. The study was conducted in accordance with the ethical principles that are consistent with the Declaration of Helsinki, International Conference on Harmonization-Good Clinical Practices, and the applicable legislation on non-interventional studies. Approval for the study protocol from an Independent Ethics Committee was obtained before the study initiation.

## Study population:

Patients of either sex, aged >18 years and who had received vildagliptin monotherapy or fixed-dose combination of vildagliptin and metformin (IR-Immediate Release) for the treatment of T2DM were identified. The patients' treatment information was sourced from the treating physician under an agreement. Patients having incomplete data files were excluded from the study. According to the investigator's discretion, unsuitable patients or patient data were also not included in the study.

## Outcomes:

The study outcomes included the evaluation of change in glycated hemoglobin (HbA1c) levels and weight changes after the treatment with vildagliptin alone or in combination with metformin therapy of various strengths. In addition, demographics of patients receiving vildagliptin alone or in combination with metformin therapy of various strengths, duration of treatment, other OADs and/or insulin and any concomitant medication received during the study period, presence of any concurrent disease and, adverse events reported within past 12 months were assessed.

## Statistical analysis:

Data were analyzed using Statistical Package for The Social Sciences (SPSS) software, version 23.0. Demographic characteristics were summarized with descriptive statistics including median and interquartile range (IQR) for continuous variables, and frequency and percentages for categorical variables. A comparison of qualitative and quantitative variables between the groups was done using the chi-square test and Mann-Whitney U test, respectively. A p-value <0.05 was considered statistically significant.

## Results

**Figure 1:**
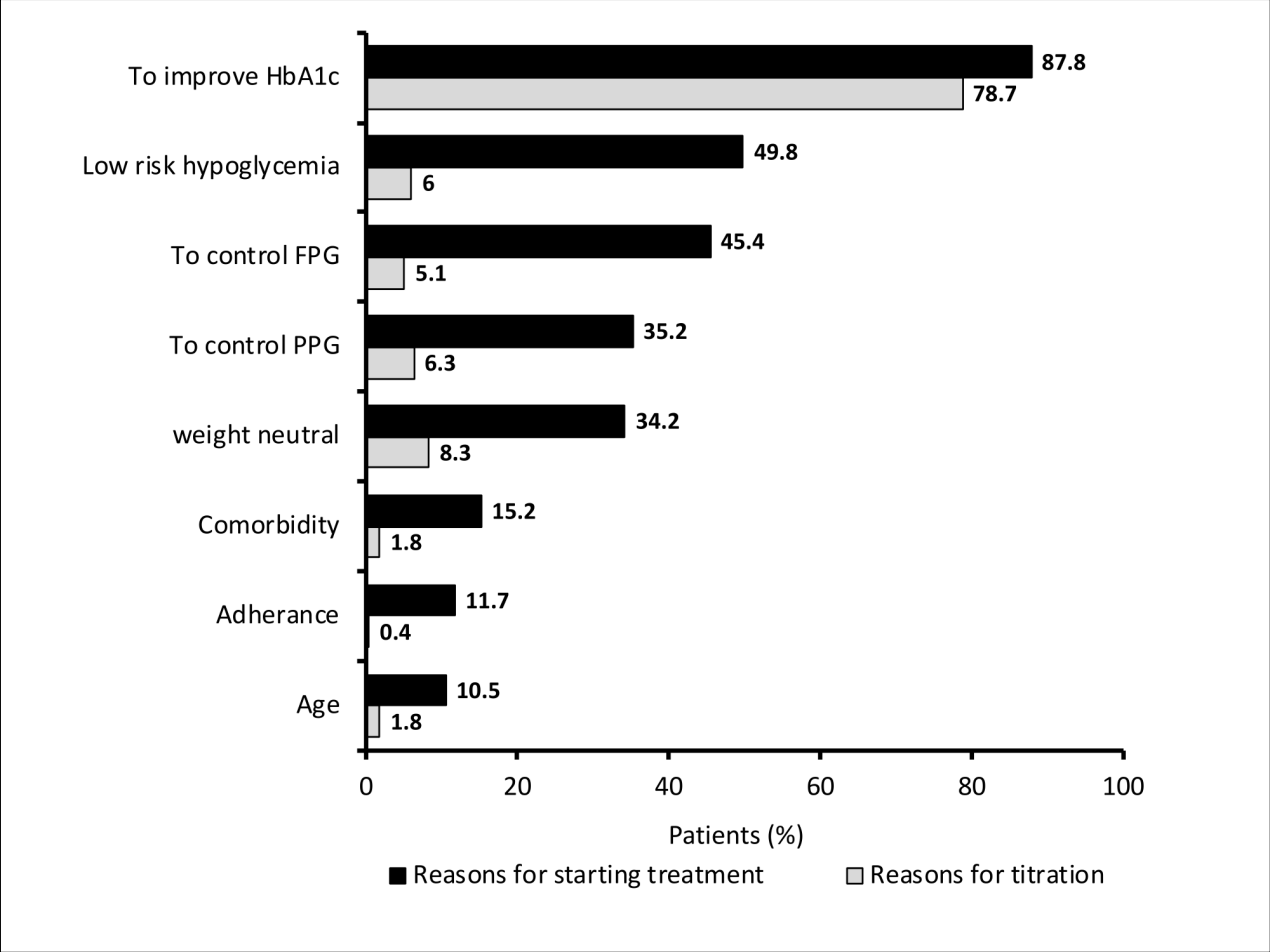
Reasons for starting vildagliptin monotherapy or vildagliptin and metformin combination and titration of dosage during study period.

**Figure 2:**
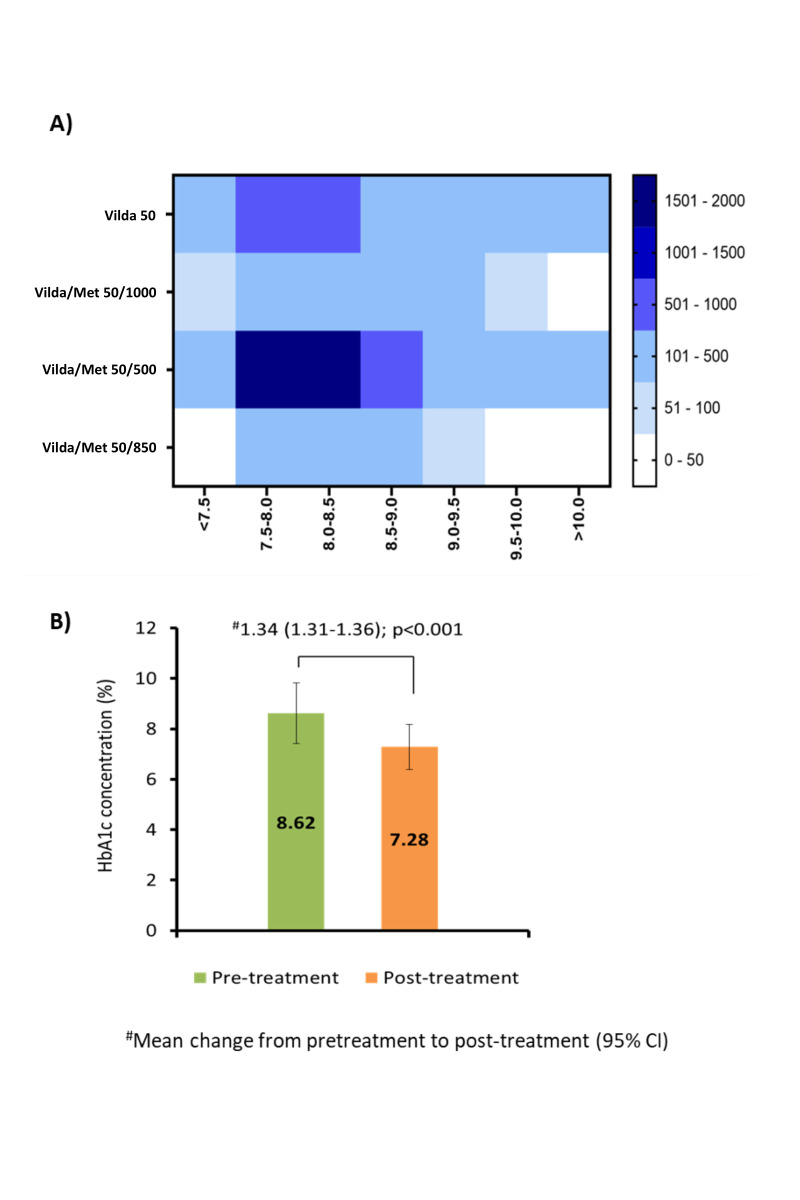
A) The trend of vildagliptin monotherapy or vildagliptin and metformin combination dosage with respect to HbA1c levels. B) Mean change in HbA1c levels from pretreatment to post treatment.

**Figure 3:**
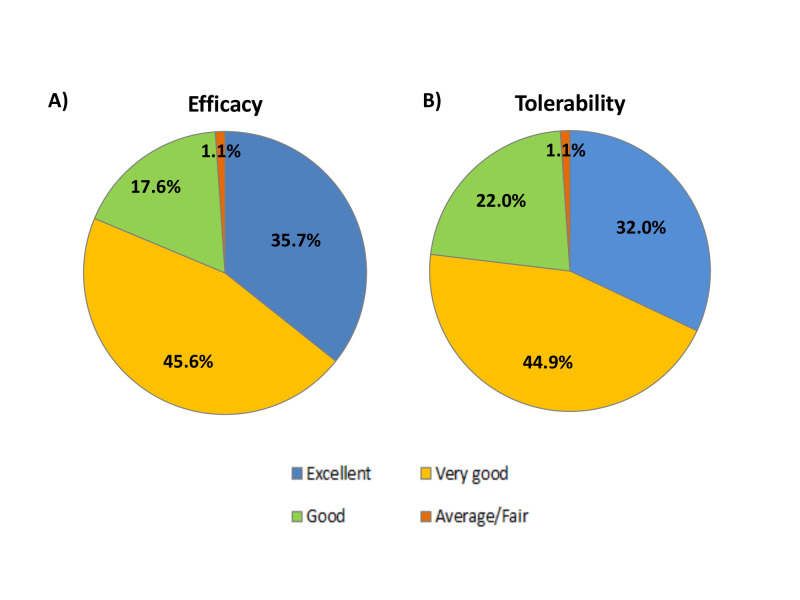
Physical global evaluation for (A) Efficacy and (B) Tolerability of the treatment.

A total of 9678 patients with T2DM were included. The median (IQR) age was 52.0 (45.0-61.0) years and the majority of patients (45.4%) were from the age group of >40 to ≤60 years. The proportion of male patients (59.1%) was higher than female patients (40.9%). The majority of the patients were enrolled in urban and semi-urban areas (83.3%). The median body mass index (BMI) of the overall population was 27.0 kg/m2. The median duration of diabetes was 60.0 months. Family history of diabetes (49.4%) and sedentary lifestyle (44.0%) were the most common risk factors observed followed by obesity (37.6%), smoking (29.6%), and emotional stress (26.3%). The most commonly observed comorbidities were hypertension (68.7%) and dyslipidemia (47.1%) while peripheral neuropathy (44.6%) and coronary artery disease (CAD) (30.6%) were the most common complications observed in the study patients (Table 1). The majority of patients (54.8%) received a combination of vildagliptin and metformin (50/500 mg) while 23.7% received vildagliptin monotherapy (50 mg), 15.1% received a combination of vildagliptin and metformin (50/1000 mg) and 6.4% received combination of vildagliptin and metformin (50/850 mg). Vildagliptin as monotherapy or combination therapy with metformin was used across a wide range of age groups from younger patients aged >18 years to elderly patients aged >60 years. Vildagliptin alone or in combination with metformin twice daily (BD) was the most frequently used dosage pattern (74.1%). The median duration of treatment (vildagliptin alone or in combination with metformin therapy) was 24.0 months (Table 2). A majority of patients (94.7%) received sulfonylureas as the concomitant anti-diabetic medication. Insulin was administered to 944 (15.7%) patients. Among the concomitant non-diabetic medications, antihypertensives (60.4%) were the most common class of drugs followed by statins (31.6%) (Table 2). The analysis revealed that the most common reason for selecting vildagliptin alone or in combination with metformin was to achieve an improvement in HbA1c levels (87.8%). Other common reasons included the control of fasting plasma glucose (45.4%) and postprandial plasma glucose (35.2%), low risk of hypoglycemia (49.8%), and weight neutrality (34.2%) (Figure 1). A total of 1969 patients required dosage titration during the treatment, the majority of them (87.5%) required dosage up-titration (Table 3). The most common reason given for titration was to improve HbA1c level (78.7%) as shown in Figure 1. Before initiating the treatment, a total of 91.5% of patients were having poor glycemic control (HbA1c ≥7.5%) where 29.6% and 28.7% of the patients were having HbA1c levels in the range of 7.5-8.0% and 8.0-8.5%, respectively (Table 3).[Figure 2A] presents the trend of vildagliptin monotherapy and combination therapy of vildagliptin and metformin with respect to HbA1c levels across the study population. The data suggested that the combination therapy of vildagliptin and metformin at 50/500 mg dose and vildagliptin monotherapy at 50 mg dose were the most commonly prescribed therapies in the patient population across a wide range of HbA1c levels from <7.5->10%. A total of 95.3% of patients achieved target glycemic control with vildagliptin monotherapy or vildagliptin and metformin combination therapy. The treatment with vildagliptin monotherapy or vildagliptin and metformin combination therapy significantly reduced the mean HbA1c levels by 1.34% [95% CI, 1.31-1.36]; p<0.001) when compared to the pre-treatment levels (8.62% vs. 7.28%) (Figure 2B). A total of 4503 (46.6%) patients experienced no change in body weight during treatment. Among 5175 (53.4%) patients who experienced body weight changes during therapy, majority of them (3551, 68.6%) had lost weight while the remaining 1624(31.4%) patients had gained weight. Of those who lost weight, more than half (N=2638) had a weight reduction in the range of 0-2 kg (Table 3). A total of 44 patients (0.4%) reported adverse events with gastritis and dyspepsia being the most common adverse events (9, each) (Table 3). Physicians rated the majority of patients as good to excellent on the global evaluation of efficacy and tolerability scale (98.9%, each) (Figure 3). observed followed by obesity (37.6%), smoking (29.6%), and emotional stress (26.3%). The most commonly observed comorbidities were hypertension (68.7%) and dyslipidemia (47.1%) while peripheral neuropathy (44.6%) and coronary artery disease (CAD) (30.6%) were the most common complications observed in the study patients (Table 1). The majority of patients (54.8%) received a combination of vildagliptin and metformin (50/500 mg) while 23.7% received vildagliptin monotherapy (50 mg), 15.1% received a combination of vildagliptin and metformin (50/1000 mg) and 6.4% received combination of vildagliptin and metformin (50/850 mg). Vildagliptin as monotherapy or combination therapy with metformin was used across a wide range of age groups from younger patients aged >18 years to elderly patients aged >60 years. Vildagliptin alone or in combination with metformin twice daily (BD) was the most frequently used dosage pattern (74.1%). The median duration of treatment (vildagliptin alone or in combination with metformin therapy) was 24.0 months (Table 2). A majority of patients (94.7%) received sulfonylureas as the concomitant anti-diabetic medication. Insulin was administered to 944 (15.7%) patients. Among the concomitant non-diabetic medications, antihypertensives (60.4%) were the most common class of drugs followed by statins (31.6%) (Table 2). The analysis revealed that the most common reason for selecting vildagliptin alone or in combination with metformin was to achieve an improvement in HbA1c levels (87.8%). Other common reasons included the control of fasting plasma glucose (45.4%) and postprandial plasma glucose (35.2%), low risk of hypoglycemia (49.8%), and weight neutrality (34.2%) (Figure 1). A total of 1969 patients required dosage titration during the treatment, the majority of them (87.5%) required dosage up-titration (Table 3). The most common reason given for titration was to improve HbA1c level (78.7%) as shown in Figure 1. Before initiating the treatment, a total of 91.5% of patients were having poor glycemic control (HbA1c ≥7.5%) where 29.6% and 28.7% of the patients were having HbA1c levels in the range of 7.5-8.0% and 8.0-8.5%, respectively (Table 3). Figure 2A presents the trend of vildagliptin monotherapy and combination therapy of vildagliptin and metformin with respect to HbA1c levels across the study population. The data suggested that the combination therapy of vildagliptin and metformin at 50/500 mg dose and vildagliptin monotherapy at 50 mg dose were the most commonly prescribed therapies in the patient population across a wide range of HbA1c levels from >7.5-<10%. A total of 95.3% of patients achieved target glycemic control with vildagliptin monotherapy or vildagliptin and metformin combination therapy. The treatment with vildagliptin monotherapy or vildagliptin and metformin combination therapy significantly reduced the mean HbA1c levels by 1.34% [95% CI, 1.31-1.36]; p<0.001) when compared to the pre-treatment levels (8.62% vs. 7.28%) (Figure 2B). A total of 4503 (46.6%) patients experienced no change in body weight during treatment. Among 5175 (53.4%) patients who experienced body weight changes during therapy, majority of them (3551, 68.6%) had lost weight while the remaining 1624(31.4%) patients had gained weight. Of those who lost weight, more than half (N=2638) had a weight reduction in the range of 0-2 kg (Table 3). A total of 44 patients (0.4%) reported adverse events with gastritis and dyspepsia being the most common adverse events (9, each) (Table 3). Physicians rated the majority of patients as good to excellent on the global evaluation of efficacy and tolerability scale (98.9%, each) (Figure 3). The median age was significantly higher in patients receiving vildagliptin 50 mg monotherapy (54.0 years) as compared to those receiving vildagliptin and metformin combinations (50/1000, 50/500, and 50/850 mg) (52.0 years, each) (p<0.001). The proportion of patients receiving combination therapy of vildagliptin and metformin 50/850 mg from age group >45-≤60 years (54.5%) was significantly higher compared to other treatment groups (p<0.001). The median (IQR) duration of diabetes was significantly higher in patients receiving a combination of vildagliptin and metformin 50/1000 mg (60.0 [36.0-108.0] months) than those receiving other therapies such as vildagliptin monotherapy 50 mg (60.0 [26.0-96.0] months), vildagliptin and metformin 50/500 mg combination therapy (60.0 [36.0-96.0] months) and vildagliptin and metformin 50/850 mg combination therapy (60.0 [36.0-84.0] months) (p<0.001). Vildagliptin or vildagliptin and metformin combinations were used in patients with T2DM with associated complications, like peripheral neuropathy, CAD, nephropathy, retinopathy, autonomous neuropathy, stroke/TIA, and PAD. In T2DM patients taking vildagliptin or vildagliptin and metformin combination therapy, the common comorbidity was hypertension and dyslipidemia observed in 64.4% to 71.1% and 45.5% to 50.7%, respectively. Obesity was observed in 42% of patients taking vildagliptin and metformin 50/1000 mg, while 25.1% to 26.3% in other dosage forms. NFALD was observed in 22.2 % of the patients taking vildagliptin 50 mg, while 3.4% to 7.2% in other dosage forms (Table 4).

## Discussion:

This real-world study documented the clinical characteristics, and treatment patterns including dosage and duration of vildagliptin monotherapy or vildagliptin and metformin combination therapy in adult patients with T2DM across 365 clinical study centers in India. In addition, it also evaluated the effect of vildagliptin therapy on glycemic control and the safety profile of patients with the T2DM continuum. A majority of the patients were from urban areas and were middle-aged with a median age of 52.0 years. A high prevalence of disease in male patients, family history of diabetes, sedentary lifestyle, and obesity were the most commonly observed risk factors of T2DM in the present study. These findings are in line with previous Indian studies. According to the Indian Council of Medical Research-India Diabetes (ICMR-INDIAB) study, age, male sex, obesity, hypertension, and family history of diabetes were the independent risk factors for diabetes in both urban and rural areas. The study also reported a higher prevalence of diabetes in urban areas, especially among low socioeconomic groups [21]. Similarly, a recent 10-year prospective cohort study from Southern India reported that age >45 years, family history of T2DM, BMI ≥25 kg/m2, and presence of obesity as the risk factors for T2DM [22]. The presence of comorbidities in Indian patients with T2DM is well established in several previous studies [5- [8]. Uncontrolled glycemia and chronic diabetes substantially contribute to the elevated risk of diabetes-associated complications. Therefore, comorbidities such as hypertension and dyslipidemia, and other complications pose a high risk of mortality for the Indian cohort of T2DM. A high prevalence of hypertension was observed in the present study population. The results corroborate with the observations from several other Indian studies reported that 20%-40% of patients have both diabetes and hypertension [22-24]. A joint consensus statement from the American and European Diabetes Associations recommends that DPP-4 inhibitor may be used if there is a need to avoid hypoglycemia or control the weight gain [10]. The use of vildagliptin has been approved in Indian patients for the treatment of T2DM as monotherapy or in combination with metformin, sulfonylureas, and thiazolidinediones, as well as with insulin [20]. In India, vildagliptin and metformin combination tablets are available in 50/500 mg, 50/850 mg, and 50/1000 mg doses [20]. The most commonly used therapy in the present study was the combination therapy of vildagliptin and metformin at 50/500 mg dose followed by vildagliptin monotherapy at 50 mg dose. Several real-world studies from India have reported the use of vildagliptin monotherapy (4%-18%) [25,26] and vildagliptin and metformin combination therapy (37%) [27] for the management of T2DM. About 70% of the present study cohort used the twice-daily formulation. In the present study, the use of vildagliptin either as monotherapy or combination therapy with metformin across a wide range of age groups (>18 years to >60 years) suggests the benefits of vildagliptin to a patient population of both younger and elder age groups. More than one-third of the patient population on vildagliptin therapy presented CAD as a complication. However, a meta-analysis of 17000 patients has provided evidence that supports the cardiovascular safety of vildagliptin [28]. Similarly, a real-world study by Williams et al. suggested that exposure to vildagliptin was not associated with an increased overall CVD risk or risk of myocardial infarction, acute coronary syndrome, stroke, and congestive heart failure when compared with other OADs [29]. In the present study, more than 70% of patients received concomitant anti-diabetic medications. Sulfonylureas were the most commonly prescribed anti-diabetic medications along with vildagliptin or vildagliptin and metformin combination therapies. A Japanese study reported that sulfonylurea (26.3%) was the second most commonly prescribed anti-diabetic drug after biguanide (54.6%) in patients with T2DM receiving vildagliptin monotherapy [17]. Several population-based studies in India have included patients who had comorbidities such as hypertension and dyslipidemia along with diabetes. Similarly, the present study also included patients with hypertension and dyslipidemia, and therefore, the most commonly prescribed concomitant medications were antihypertensives and statins [16, 20]. The present study suggested that monotherapy of vildagliptin or combination therapy with metformin showed greater improvements in the mean HbA1c and greater reduction in the body weight. More than 90% of the patients had uncontrolled glycemic levels before treatment and good glycemic control achieved by 95.3% of patients after the treatment. These observations suggest the clinical effectiveness of vildagliptin monotherapy or vildagliptin and metformin combination therapy in achieving glycemic control and weight neutralization. A meta-analysis of 58 randomized controlled trials, DPP4-inhibitors, vildagliptin 50 mg BD, and linagliptin 10 mg QD, suggested a significant lowering effect on the glycemic indices in comparison to the placebo [30]. The 5-year long trial results suggest early intervention with vildagliptin plus metformin provides significant continuing benefits compared to the initial metformin monotherapy used for patients with newly diagnosed T2DM [31]. In the post-hoc analysis of an observational real-world EDGE study, Wangnoo et al. assessed the safety and efficacy of vildagliptin in combination with another OAD in 11,057 Indian patients with T2DM. Vildagliptin and metformin combination was used in more than 70% of the study population. The HbA1c reduction was in favor of vildagliptin usage, achieved in 68.5% in the vildagliptin cohort compared to the comparator cohort (56.8%), with an unadjusted OR of 1.65 (95% CI: 1.53, 1.79; p < 0.0001) [18]. In previous studies, vildagliptin or vildagliptin and metformin combination therapy was well tolerated [18,31]. These findings are in line with the current study results that have shown a smaller number of patients experiencing adverse events with these treatments. Physicians' global evaluation of efficacy and tolerability showed a majority of patients on a good to excellent scale (98.9%). Evidence from Indian literature suggests that, of all the combination of OADs, the combination of metformin and vildagliptin was prescribed by the majority of the physicians [27,32]. These observations support the use of vildagliptin as an add-on therapy to metformin and are the preferred choice of therapy by physicians in Indian settings. The present study has several limitations. Due to the retrospective nature of this study, several parameters such as the antidiabetic regimens used before vildagliptin or vildagliptin/metformin combination therapy and time of the previous visit could not be captured, which may have an indirect effect on the overall study results.

## Conclusion

Vildagliptin with or without metformin was an effective therapy in reducing HbA1c that helped in achieving target glycemic control and was well tolerated in Indian patients with the T2DM continuum. The use of vildagliptin therapy in patients with comorbidities (hypertension and dyslipidemia), complications (peripheral neuropathy, CAD, nephropathy, and retinopathy), different age groups (younger to elderly patients), and physician acceptance suggests wide use of vildagliptin for each subgroup of the diabetic continuum in Indian settings.

## Figures and Tables

**Table 1:** Patient demographics and treatment related observations.

Parameters	Values (N=9678)*
Age (years), [n=9656]	52.0 (45.0-61.0)
Age group (years), n (%) [n=9656]	
>18-≤45	2701 (28.0)
>45-≤60	4386 (45.4)
>60	2569 (26.6)
Sex, n (%) [n=9422]	
Men	5568 (59.1)
Women	3854 (40.9)
Locality, n (%) [n=7519]	
Urban/Semi-urban	6263 (83.3)
Rural/Semi-rural	1256 (16.7)
Height (cm), [n=9297]	163.0 (157.0-169.0)
Weight (kg), [n=9574]	71.0 (65.0-79.0)
BMI (kg/m2), [n=9295]	27.0 (24.4-29.8)
Duration of diabetes (months), [n=9544]	60.0 (36.0-96.0)
Biochemical investigations	
FPG (mg/dL), [n=6402]	117.0 (103.0-133.0)
PPG (mg/dL), [n=6222]	170.0 (148.0-198.0)
Total cholesterol (mg/dL), [n=3134]	175.0 (154.0-200)
HDL-C (mg/dL), [n=2963]	42.0 (37.0-47.0)
LDL-C (mg/dL), [n=2896]	109.0 (90.0-131.0)
Triglyceride (mg/dL), [n=2830]	157.0 (128.0-192.0)
Serum creatinine (mg/dL), [n=2952]	0.9 (0.8-1.1)
Urine albumin (mg/g), [n=414]	23.0 (12.6-31.0)
Risk factors [n=8928], n (%)	
Family history of DM	4478 (49.4)
Sedentary lifestyle	3985 (44.0)
Obesity	3406 (37.6)
Smoking	2685 (29.6)
Emotional stress	2386 (26.3)
Intake of excess salt	1796 (19.8)
Alcohol consumption	1264 (13.9)
Tobacco chewing	851 (9.4)
Complications [n=3958], n (%)	
Peripheral neuropathy	1764 (44.6)
CAD	1213 (30.6)
Nephropathy	865 (21.9)
Retinopathy	737 (18.6)
Autonomous neuropathy	546 (13.8)
Stroke/TIA	170 (4.3)
PAD	121 (3.1)
Others	39 (0.9)
Comorbidities [n=7752], n (%)	
Hypertension	5326 (68.7)
Dyslipidemia	3650 (47.1)
Obesity	2163 (27.9)
NAFLD	347 (4.5)
Data shown as median (IQR), unless otherwise specified. *N=9678, unless otherwise specified. BMI, body mass index; CAD, coronary artery disease; DM, diabetes mellitus; FPG, fasting plasma glucose; HDLhigh density lipoprotein; IQR, interquartile range; LDL, low density lipoprotein; NAFLD, non-alcoholic fatty liver disease; PAD, peripheral artery disease; PPG, postprandial plasma glucose; TIA - transient ischemic attack.

**Table 2:** Observations related to various medications received across the study population.

Parameters	Values (N=9678)*
Treatment pattern of drug dosage (mg)	
Vildagliptin and Metformin (50/500)	5307 (54.8)
Vildagliptin (50)	2281 (23.7)
Vildagliptin and Metformin (50/1000)	1466 (15.1)
Vildagliptin and Metformin (50/850)	624 (6.4)
Frequency of dose [n=8957]	
OD	2328 (25.9)
BD	6629 (74.1)
Duration of treatment (months), median (IQR) [n=8830]	24.0 (12.0-36.0)
Concomitant anti-diabetic medications	6000 (61.9)
Sulfonylureas	5684 (94.7)
Insulin	944 (15.7)
SGLT 2 l	890 (14.8)
Thiazolidinedione	848 (14.1)
AGIs	721 (12.0)
GLP1 agonist	27 (0.4)
Concomitant non-diabetic medications	
Antihypertensive	5850 (60.4)
Statins	3061 (31.6)
Neuropathic pain	183 (1.9)
Antiplatelet	179 (1.8)
Others	1766 (18.2)
Data shown as n (%), unless otherwise specified. *N=9678, unless otherwise specified. AGIs, alpha-glucosidase inhibitors; BD, twice a day; GLP1, glucagon-like peptide-1; IQR, interquartile range; OD, once a day; BD, twice a day; SGLT 2 I, sodium-glucose co-transporter-2 inhibitor. Neuropathic pain included anti-anxiety drugs and non-steroidal anti-inflammatory drugs. Others, patients who were on concomitant non-diabetic medication including antacids, antibiotics, anticoagulants, anticonvulsants, anti-emetics, antihistamines, anti-malarials, thyroxine, vitamins and multivitamins.

**Table 3:** Observations related to weight alterations, glycemic control, and adverse events.

Parameters	Number of patients (%)
Dose titration of vildagliptin or vildagliptin and metformin combination [n=1969]	
Dosage up titration	1724 (87.5)
Dosage down titration	245 (12.5)
HbA1c level before treatment initiation [n=9328]	
<7.5	795 (8.5)
7.5-8.0	2763 (29.6)
8.0-8.5	2681 (28.7)
8.5-9.0	1460 (15.6)
9.0-9.5	835 (9.0)
9.5-10.0	462 (5.0)
>10.0	332 (3.6)
Patients with weight changes during the therapy [n=5175]	
a) Weight gain (kg)	
0-2	1101 (21.3)
4-Feb	455 (8.8)
>4	68 (1.3)
b) Weight loss (kg)	
0-2	2638 (50.9)
4-Feb	754 (14.6)
>4	159 (3.1)
Patients achieving the glycemic goal [n=9678]	9223 (95.3)
Number of adverse events reported [n=44]	
Gastritis	9
Dyspepsia	9
GI disease	7
Giddiness	6
Nausea	2
Diarrhea	2
Hypoglycemia	2
Others	7
Data shown as n (%). *N=5695, unless otherwise specified. FPG, fasting plasma glucose; GI, gastrointestinal; HbA1c, glycosylated hemoglobin; PPG, postprandial plasma glucose. Other adverse events include abdominal discomfort and inadequate bowel movement, acidity, and constipation.

**Table 4:** Treatment wise patient demographics observations.

Characteristics	Group A Vildagliptin 50 mg (N=2281)*	Group B Vildagliptin and Metformin 50/1000 mg (N=1466)**	Group C Vildagliptin and Metformin 50/500 mg (N=5307)#	Group D Vildagliptin and Metformin 50/850 mg (N=624)##	P value
Age (years), median (IQR)	[n=2272] 54.0 (46.0-63.0)	[n=1464] 52.0 (43.0-62.0)	[n=5296] 52.0 (44.0-60.0)	52.0 (46.0-60.0)	0.001a, b, 0.004c, 0.080d, 0.882e, 0.287f
Age group (years)					
>18-≤45	550 (24.2)	433 (29.6)	1568 (29.6)	150 (24.0)	<0.001
>45-≤60	984 (43.3)	622 (42.5)	2440 (46.1)	340 (54.5)	
>60	738 (32.5)	409 (27.9)	1288 (24.3)	134 (21.5)	
Sex	[n=2215]	[n=1416]	[n=5177]	[n=614]	
Men	1276 (57.6)	848 (59.9)	3084 (59.6)	360 (58.6)	0.042
Women	939 (42.4)	568 (40.1)	2093 (40.4)	254 (41.4)	
BMI (kg/m2), median (IQR)	[n=2174] 26.1 (23.7-29.0)	[n=1418] 28.3 (25.5-31.1)	[n=5101] 26.7 (24.3-29.6)	[n=602] 27.8 (25.0-30.7)	<0.001a, b, c, d, e, f
Location	[n=1761]	[n=1118]	[n=4137]	[n=503]	
Urban	1430 (81.2)	945 (84.5)	3496 (84.5)	392 (77.9)	<0.001
Rural	331 (18.8)	173 (15.5)	641 (15.5)	111 (22.1)	
Duration of diabetes (months), median (IQR)	[n=2250] 60.0 (26.0-96.0)	[n=1451] 60.0 (36.0-108.0)	[n=5283] 60.0 (36.0-96.0)	[n=622] 60.0 (36.0-84.0)	0.115a, 0.063b, 0.213c, 0.001d, 0.022e, 0.746f
Complications	[n=879]	[n=569]	[n=2179]	[n=331]	
Peripheral neuropathy	406 (46.2)	271 (47.6)	981 (45.0)	106 (32.0)	<0.001
CAD	300 (34.1)	202 (35.5)	618 (28.4)	93 (28.1)	0.001
Nephropathy	165 (18.8)	145 (25.5)	479 (22.0)	76 (23.0)	0.023
Retinopathy	96 (10.9)	104 (18.3)	460 (21.1)	77 (23.3)	<0.001
Autonomous neuropathy	116 (13.2)	85 (14.9)	306 (14.0)	39 (11.8)	0.546
Stroke/TIA	40 (4.6)	30 (5.3)	91 (4.2)	9 (2.7)	0.316
PAD	20 (2.3)	34 (6.0)	61 (2.8)	6 (1.8)	<0.001
Comorbidities	[n=1693]	[n=1243]	[n=4268]	[n=548]	
Hypertension	1126 (66.5)	800 (64.4)	3033 (71.1)	367 (68.7)	<0.001
Dyslipidemia	859 (50.7)	587 (47.2)	1940 (45.5)	264 (48.2)	0.003
Obesity	425 (25.1)	522 (42.0)	1072 (25.1)	144 (26.3)	<0.001
NAFLD	77 (22.2)	89 (7.2)	146 (3.4)	35 (6.4)	<0.001
Duration of treatment (months), median (IQR)	[n=2002] 24.0 (12.0-36.0)	[n=1359] 24.0 (12.0-36.0)	[n=4886] 24.0 (12.0-36.0)	[n=583] 24.0 (12.0-36.0)	<0.001a, b, 0.002c, 0.081d, 0.154e, 0.693f
HbA1c level (%)					
Before treatment initiation	[n=1144] 8.3 (7.8-9.0)	[n=603] 8.5 (8.0-9.0)	[n=2339] 8.4 (7.9-9.0)	[n=249] 8.5 (8.1-8.9)	<0.001a, b, 0.001c, 0.073d, 0.984e, 0.184f
After treatment	[n=1305] 7.1 (6.7-7.6)	[n=657] 7.1 (6.9-7.6)	[n=2676] 7.1 (6.8-7.6)	[n=279] 7.1 (6.9-7.6)	0.074a, 0.111b, 0.322c, 0.479d, 0.706e, 0.900f
Data shown as n (%), unless otherwise specified. *N=2281; **N=1466; #N=5307; ##N=624, unless otherwise specified.
BMI, body mass index; CAD, coronary artery disease; IQR, interquartile range; NAFLD, nonalcoholic fatty liver disease; PAD, peripheral artery disease; TIA, transient ischemic attack.
a group A vs B; b group A vs C; c group A vs D; d group B vs C; e group B vs D; f group C vs D.
